# Monocytes, Macrophages, and Their Potential Niches in Synovial Joints – Therapeutic Targets in Post-Traumatic Osteoarthritis?

**DOI:** 10.3389/fimmu.2021.763702

**Published:** 2021-11-04

**Authors:** Patrick Haubruck, Marlene Magalhaes Pinto, Babak Moradi, Christopher B. Little, Rebecca Gentek

**Affiliations:** ^1^ Centre for Orthopaedics, Trauma Surgery and Spinal Cord Injury, Trauma and Reconstructive Surgery, Heidelberg University Hospital, Heidelberg, Germany; ^2^ Raymond Purves Bone and Joint Research Laboratory, Kolling Institute, Institute of Bone and Joint Research, Faculty of Medicine and Health University of Sydney, Royal North Shore Hospital, St. Leonards, NSW, Australia; ^3^ Centre for Inflammation Research & Centre for Reproductive Health, University of Edinburgh, Edinburgh, United Kingdom; ^4^ Clinic of Orthopaedics and Trauma Surgery, University Clinic of Schleswig-Holstein, Kiel, Germany

**Keywords:** osteoarthritis, monocyte - macrophage, inflammation, niche, native immune functions, synovitis, immunomodulation

## Abstract

Synovial joints are complex structures that enable normal locomotion. Following injury, they undergo a series of changes, including a prevalent inflammatory response. This increases the risk for development of osteoarthritis (OA), the most common joint disorder. In healthy joints, macrophages are the predominant immune cells. They regulate bone turnover, constantly scavenge debris from the joint cavity and, together with synovial fibroblasts, form a protective barrier. Macrophages thus work in concert with the non-hematopoietic stroma. In turn, the stroma provides a scaffold as well as molecular signals for macrophage survival and functional imprinting: “a macrophage niche”. These intricate cellular interactions are susceptible to perturbations like those induced by joint injury. With this review, we explore how the concepts of local tissue niches apply to synovial joints. We introduce the joint micro-anatomy and cellular players, and discuss their potential interactions in healthy joints, with an emphasis on molecular cues underlying their crosstalk and relevance to joint functionality. We then consider how these interactions are perturbed by joint injury and how they may contribute to OA pathogenesis. We conclude by discussing how understanding these changes might help identify novel therapeutic avenues with the potential of restoring joint function and reducing post-traumatic OA risk.

## Introduction

Osteoarthritis (OA) is the most common joint disorder (1). Its prevalence is expected to increase further (2) due to rising societal levels of ageing, obesity and injury, key risk factors for OA. While the disease commonly affects knees, hips, hands and feet, OA of the knee accounts for more than 80% of the disease burden (1, 3). The knee is particularly susceptible to injury, with approximately 40% of patients that suffer a traumatic knee injury developing so-called “post-traumatic” (pt)OA, and surgical reconstruction and restoration of joint biomechanics insufficient to prevent its development (4). Treatment options for OA are very limited, and there is a particular need for effective preventive and disease modifying drugs (DMD). This is highlighted by clinical data showing comparable disease burden at diagnosis but significantly higher burden 6 months later in OA compared to rheumatoid arthritis (RA) patients (5). Owing to this paucity of treatment options and the high and rising prevalence, OA contributes substantially to the global burden of disease. In a 2015 survey, OA was identified as the second most prevalent cause for years lived with disability (2), highlighting the impact OA has on both individuals and society (2).

Although the name osteoarthritis implies an inherent inflammatory process (6), it was historically believed that OA had purely biomechanical causes (7). Indeed, OA was regarded a disease of the elderly, inevitably caused by years of “wear and tear”. Breaking with this previously held view, we now know that OA development involves a complex active biological response with local interaction between joint tissues and their resident cells, and these with systemic mediators. This includes an inflammatory response (8) that is accompanied by complex metabolic changes, which contribute to cartilage degradation and activation of bone remodeling (9). Although innate immune cells, and monocytes and macrophages in particular have been implicated (7, 10, 11), the exact nature of the inflammatory response in OA, its underlying mechanisms and its relative contribution to onset or progression of structural pathology and symptoms remain incompletely resolved (12).

Much like OA etiology, our understanding of the complex development and functional heterogeneity of macrophages and monocytes as well as their interactions within local tissue “niches” has dramatically changed in recent years. It is now firmly established that the long-held paradigm of discrete, polarized monocyte and macrophage activation states is an oversimplification of what in reality is a spectrum of cell states. Likewise, it is now recognized that macrophages established from fetal progenitors can persist in adult tissues, and that many macrophages self-maintain independently of monocytes (13–16). Lastly, we are beginning to appreciate that macrophages engage in bidirectional crosstalk with other cell types within their local niches, interactions that are of mutual benefit and implicate macrophages as gatekeepers of tissue function (17, 18). While much remains to be learned and confirmed, these concepts developed in other organs and tissues appear to also apply to macrophages in joints (19, 20). Indeed, macrophages found in the healthy synovium are predominantly monocyte-independent, and they protect and contribute to joint homeostasis in several ways, including barrier formation, clearance of debris and even lubrication (20). Under inflammatory conditions, such as may occur with joint injury however, monocytes are recruited to the affected joint and can differentiate into macrophages, which retain a more inflammatory phenotype (21). These joint macrophage populations thus not only differ in their origins, but also exert distinct functions (22, 23).

Modifying the developmental, functional and *in situ* dynamics of joint macrophages and monocytes might therefore represent an attractive avenue for novel therapeutic approaches in OA. This may be particularly relevant in ptOA, where causal initiation and subsequent temporal changes in monocytes, macrophages and their activation with disease onset and progression may be targeted. This review aims to explore this notion, with a focus on the synovial rather than osseous joint tissue niche. We will summarize experimental and clinical studies on macrophages and monocytes in healthy and diseased joints and interpret these in the context of current paradigms of myeloid biology. Our emphasis in this review is on joint injury and ptOA, as this represents the major OA phenotype studied in pre-clinical research, and as noted above, it has the most well demarcated disease stages and thus potentially the broadest therapeutic opportunity. In doing so, we hope to bridge persisting gaps between bench and bedside and highlight research questions with the potential to pave the way towards better treatment options for ptOA, but also other OA phenotypes more broadly.

## Macro- and Microanatomy of the Knee Joint

**Figure 1:**
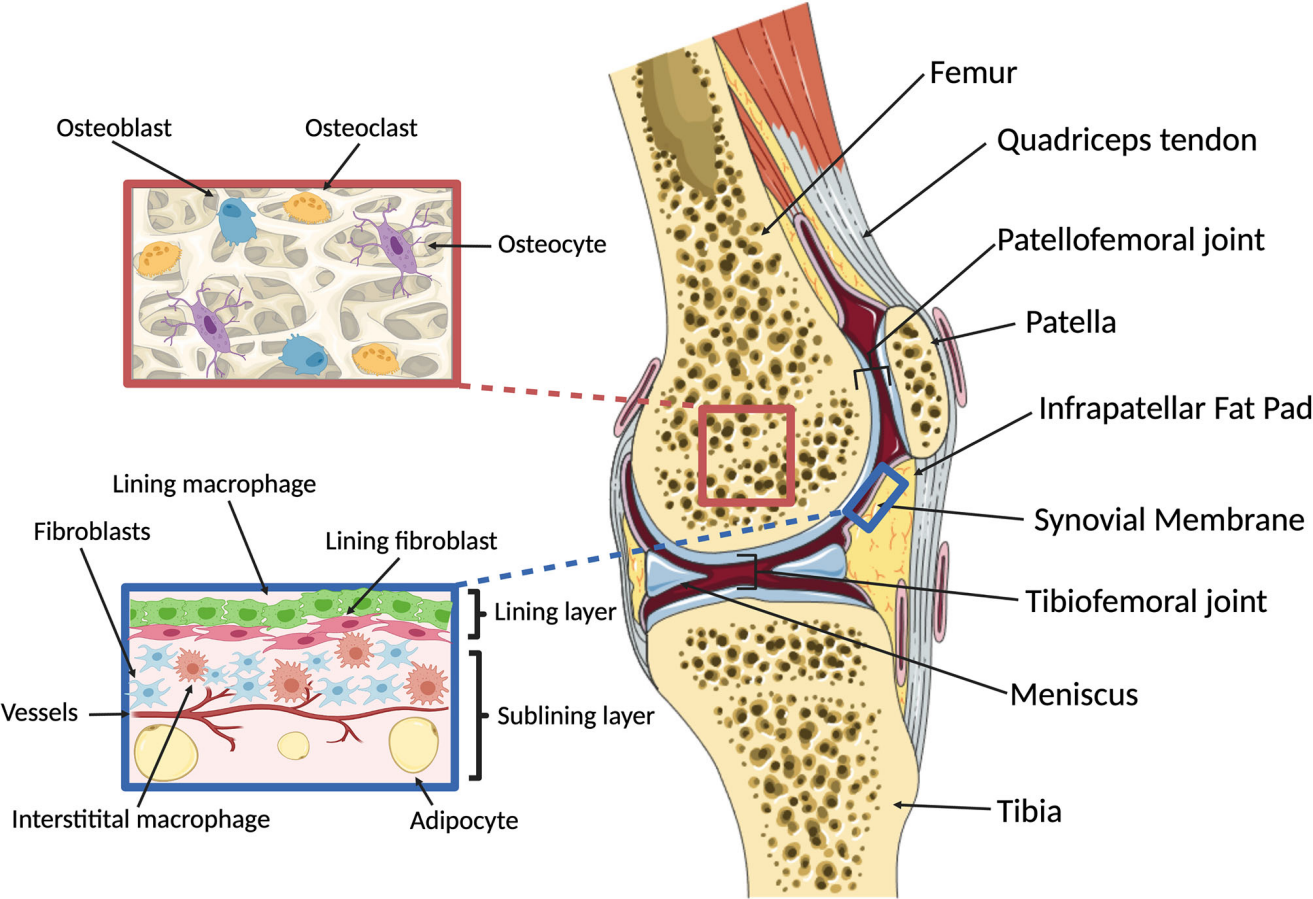
Overview of knee macro- and microanatomy. (Right) Sagittal cut through a human knee. The femur and tibia articulate in the tibiofemoral joint, with two fibrocartilaginous menisci serving to provide rotational and anterior-posterior stability and load distribution. The patella is a hypomochlion (pivot point) for the quadriceps tendon that articulates with the femur in the patellofemoral joint. The intra-articular components of the osseous structures are covered by cartilage, enabling low-friction bearing. Joint function and stability are maintained by ligaments and the joint capsule. The inner surface of the joint capsule is lined with the synovial membrane, which is accompanied by adipose tissues located intra-articularly and extra-synovially, including the infrapatellar fat pad. (Left, top) Microanatomy of subchondral bone. The cellular components of bone include osteoblasts, osteoclasts and osteocytes that dynamically respond to changes in mechanical loading and potentially communicate with the overlying cartilage *via* soluble signals. (Left, bottom) Microanatomy of the synovial membrane. The synovium comprises three layers: the intimal lining layer which consists of macrophages and fibroblasts that together form a semi-permeable protective barrier; the vascularized subintimal or sublining layer which contains interstitial macrophages and fibroblasts as well as adipocytes; and an outer fibrous stromal layer forming the joint capsule (not shown). Created with BioRender.com and smart.servier.com.

Synovial joints provide critical motion segments that allow low friction movement between rigid (osseous) skeletal components. They enable diverse and essential bio-mechanical functions ranging from fine movements of arms, hands and fingers through to walking, running and jumping. The knee represents an anatomically complex example of a joint (**Figure 1**) that enables locomotion in a variety of terrains, while minimizing muscular energy requirements and absorbing and redistributing forces that originate from the contact between the walking surface and the foot (24). Its main osseous components are the femur, tibia and patella, that articulate in two locations: the tibiofemoral and patellofemoral joints. The menisci, two C-shaped fibro-cartilaginous structures, absorb and distribute load between the femoral and tibial surfaces. Together with a multitude of extra- and intra-articular ligaments and the fibrous joint capsule, the menisci also provide stability in flexion/extension and rotation, enabling the unique biomechanical function of the knee (25). As in all joints, the osseous surfaces in the knee are covered by hyaline cartilage, a sparsely cellular, deformable connective tissue matrix with key components of collagen type II and highly hydrated proteoglycans. Cartilage is heterogenous and can be broadly divided into three zones based on depth from the surface. These have distinct composition, biomechanical properties and functions (26). Chondrocytes make up about 2% of the articular cartilage volume (27) and are responsible for the maintenance and repair of the cartilage extracellular matrix. They are highly specialized cells derived from mesenchymal stem cells that have limited potential for replication *in situ*, but can react to a plethora of mechanical and molecular stimuli (26). The knee also harbors several adipose tissues. These are located intra-articularly and extra-synovially, and include the infrapatellar fat pad, which can be considered a highly specialized compartment in the sublining interstitial tissue [in humans known as Hoffa’s fat pad (28)]. Beyond filling the space in the joint cavity and absorbing shock, adipose tissues also secrete cytokines and adipokines (29, 30) and are therefore potent immune-modulators. It is also believed that the infrapatellar fat pad engages in intimate crosstalk with the synovial membrane, a specialized connective tissue that lines the inner surface of the joint capsule (31). The synovial membrane consists of three layers: The intimal lining layer is found closest to the joint cavity and consists mainly of macrophages (“type A cells”) and fibroblasts (“type B cells”) that show low degrees of cell division (31). Beneath this is the vascularized subintimal layer, also referred to as sublining interstitial tissue, and finally a fibrous stromal layer forming the joint capsule. The synovial membrane maintains joint homeostasis by providing lubrication and nutrition to the cartilage. It also forms a semi-permeable protective barrier that controls the molecular traffic in and out of the joint (32) and renders the synovial cavity relatively immune-privileged (20). Because of its critical role in joint homeostasis, this review will largely focus on monocyte and macrophage biology of the synovial membrane, including its sublining interstitial layer.

## A Revised View of Monocyte and Macrophage Biology

Many historically held views of monocyte and macrophage biology have been overhauled in recent times, including their phenotypic and functional heterogeneity, developmental dynamics as well as their crosstalk and functional interdependence with other cell types in the same tissue microenvironment.

### Monocyte and Macrophage Development

It was previously believed that the key (if not sole) function of monocytes was to produce macrophages, and that in turn, all macrophages found in peripheral tissues originate exclusively from monocytes (33). Elegant studies exploiting genetic fate mapping have since shown that most tissue-resident macrophages are in fact of fetal origin and self-maintain in adult tissues independently of bone marrow (BM)-derived monocytes (33). Indeed, macrophages colonize tissues concomitantly with their development in what appears to be a demand-driven way. They are generated from successive, but overlapping waves of hematopoietic progenitors produced at distinct anatomical sites (34). The majority of fetal macrophages originate from erythro-myeloid progenitors (EMP) produced in the extra-embryonic yolk sac (15, 16). EMP are fetal-restricted progenitors that differentiate into macrophages either directly or *via* fetal liver intermediates, but as an uncommitted entity do not persist into adulthood.

This new paradigm of predominantly fetal origins of tissue macrophages notwithstanding, monocytes can still complement tissue phagocyte compartments on demand (33). While this applies to some tissues at homeostasis (e.g. skin and gastro-intestinal tract), it is particularly true and important in inflammatory conditions. Importantly, in both scenarios, monocytes themselves have a number of key effector functions (35).

Monocytes differentiate from BM hematopoietic stem cells (HSC) in a strictly hierarchical, tree-like maturation process (35). They share a common progenitor with dendritic cells (DCs) known as “monocyte-macrophage DC progenitor” (MDP) (36, 37), which gives rise to a monocyte-committed intermediate, designated the “common monocyte progenitor” (cMoP) (38). The downstream “transitional pre-monocytes” (TpMos) (38, 39) are believed to be the final intermediate stage in monocyte differentiation (39). They are capable of rapid proliferation and express high levels of C-X-C motif chemokine Receptors (CXCR) 4, which anchors them to the BM. Based on differential expression of Lymphocyte antigen 6C (Ly6C), CX3CR1 and C-Chemokine Receptor type 2 (CCR2) in mice (40) or Cluster of Differentiation (CD)14 and CD16 in humans (37), mature monocytes can be broadly classified into classical (mice: Ly6C^high^ CX3CR1^low^ CCR2^high^; humans: CD14^high^ CD16^-^) and non-classical monocytes (mice: Ly6C^low^ CX3CR1^high^ CCR2^low^; humans: CD14^low^ CD16^+^) (35, 41–44). This binary classification is now widely established (45) and has more recently been backed up by extensive high-dimensional studies, the latter also revealing a previously underappreciated heterogeneity (46). A third monocyte population with an intermediate phenotype is exclusive to humans (CD14^+^ CD16^+^) (35). Classical and non-classical monocytes differ in a number of features, including their relative abundance and the regulatory mechanisms governing their retention in and egress from the BM (47). The mature monocyte compartment in the BM is vastly predominated by Ly6C^high^ monocytes, which downregulate CXCR4 (48, 49) and highly express CCR2 (50, 51), collectively enabling their egress from the BM. Ly6C^low^ monocytes, on the other hand, only express very low levels of CCR2, and while still under investigation, Sphingosine-1-Phosphate Receptor 5 (S1PR5) signaling has been implicated in orchestrating their BM egress (52).

Once released into the blood stream, classical Ly6C^high^ monocytes have a relatively short half-life lasting for a mere 20-24 hours in mice (53–55), whereas their non-classical counterparts are slightly longer-lived with a half-life of around 2 days in mice and 7 days in humans (53). The two populations are also developmentally connected: lineage tracing indicating that Ly6C^low^ monocytes originate from aging Ly6C^high^ monocytes (41, 53), a gradual conversion that is dependent on Nuclear Receptor subfamily 4 group A member 1 (NR4A1) signaling (56) and involves direct cellular contact with endothelial cells and Notch signaling (57, 58). Similar mechanisms appear to be at play in human monocytes (59, 60). At homeostasis, Ly6C^low^ monocytes do not normally extravasate but instead patrol the luminal side of the endothelium (61). They roll along the vascular endothelium, independent of the direction of the blood flow, *via* CX3CR1, β2 integrin (58, 62) and interactions between Lymphocyte Function-associated Antigen-1 (LFA-1) and IntraCellular Adhesion Molecule 1 (ICAM1) and ICAM2 (58, 62). They have thus been considered the “tissue-resident” macrophages of blood vessels. In non-homeostatic conditions, Ly6C^low^ monocytes are thought to promote resolution of inflammation, however, they can also contribute to autoimmunity and chronic inflammatory diseases (58), as we will discuss further below. Intriguingly, experiments using bleomycin-induced lung fibrosis in mice identified an alternative pathway to Ly6C^low^ monocytes, consisting of a separate progenitor referred to as a “Segregated-nucleus-containing atypical Monocyte (SatM)”, whose production depends on the transcription factor C/EBPβ (63). Whether this pathway is relevant to other pathologies remains to be determined.

Unlike their non-classical counterparts, Ly6C^high^ monocytes do traffic into peripheral tissues even at steady state (64). In tissues that (partially) rely on homeostatic renewal from the BM, such as the skin and gastro-intestinal tract (65, 66), the majority of recruited Ly6C^high^ monocytes gradually differentiate into macrophages, a process phenotypically characterized as a “monocyte waterfall” (67). These macrophages are functionally imprinted in response to local cues that superpose tissue-specific identity onto a transcriptional core lineage program (68, 69). Provided monocytes encounter a homeostatic environment and are allowed sufficient time in the tissue, monocyte-derived macrophages are phenotypically, transcriptomically and epigenetically indistinguishable from pre-existing tissue-resident macrophages (70, 71). However, this is not the case following inflammation or other insults resulting in perturbed homeostasis, which might have important functional implications. Indeed, different and sometimes even opposing roles have been reported for developmentally distinct macrophages in conditions like cancer (72–74) and stroke (75), and this might also be the case in joint pathology, as we will discuss below.

### Monocyte Effector Functions

In addition to representing an “on-demand” source for macrophages, monocytes also have important effector functions in their own right. Indeed, a fraction of classical monocytes recruited at steady state maintains their monocytic phenotype with minimal transcriptional changes (76). In the parenchyma of non-lymphoid organs like the skin, lung, and heart (43, 66), they contribute to immune surveillance. During sterile inflammatory responses, as would occur following closed traumatic knee injury, Ly6C^high^ and Ly6C^low^ monocytes are recruited in a highly orchestrated manner facilitated by differential chemokine release. Under such conditions, Ly6C^low^ monocytes have primarily been attributed beneficial, anti-inflammatory roles. In the ischemic heart and kidney for example, deficiency in Ly6C^low^ monocytes results in higher inflammatory levels and impaired restoration of organ function (77–79). In line with this, Ly6C^low^ monocytes predominantly produce anti-inflammatory mediators like Interleukin (IL)-10 (80, 81) as well as Vascular Endothelial Growth Factor (VEGF) and other pro-angiogenic factors (82), as observed during spinal cord injury and myocardial infarction (82), respectively.

Somewhat contradictory evidence exists regarding the role of Ly6C^high^ monocytes. Historically, these classical monocytes have been recognized as potent pro-inflammatory effector cells. Indeed, CCR2 knockout mice, which are largely deficient in classical monocytes in the periphery, show decreased levels of IL-1β and Tumor Necrosis Factor (TNF)-α and an increase in the anti-inflammatory cytokines IL-4, IL-5 and IL-13 at the site of inflammation during cerebral ischemia (83). Ly6C^high^ monocytes also show high levels of reactive oxygen species, TNF-α and IL-6 (84) in the context of liver ischemia reperfusion injury (84). In line with this pro-inflammatory phenotype, Ly6C^high^ monocytes mediate tissue damage in the ischemic liver as well as the heart following myocardial infarction, and contribute to progression of atherosclerosis (84–87). At the same time however, Ly6C^high^ monocytes have also been implicated in regression of atherosclerosis (88), although this may be attributable to anti-inflammatory effects of monocyte-derived macrophages, rather than a true monocyte effector function. Nonetheless, these findings collectively suggest that instead of being globally pro- and anti-inflammatory, classical and non-classical monocytes differentially shape the local inflammatory response *via* the tailored production of cytokines and other cellular mediators. Although similarities exist between tissues and insults, their exact trafficking patterns and effector functions appear to be context-dependent, and therefore need to be delineated specifically in the homeostatic, injured and OA joint.

### Tissue Adaptation and Activation of Monocytes and Macrophages

Monocytes and macrophages dynamically respond to a variety of cues in their microenvironment, which shape their local tissue adaptation and activation state. Consequently, although they share a lineage-defining core transcriptomic signature, macrophages in different tissues are transcriptionally, phenotypically and functionally very diverse (70, 71, 89). The core macrophage program is initiated in committed fetal progenitors or BM-derived monocytes and driven by lineage-determining transcription factors (68–71).

Acquisition of tissue-specific identity and function is subsequently orchestrated by additional transcription factors in response to signals present in the local microenvironment (69). In the spleen, for example, heme from senescent red blood cells induces expression of the transcription factor SPI-C, which in turn activates a transcriptional program inducing differentiation of red pulp macrophages (90). Experimental data from adoptive transfer experiments demonstrate that exposure to different environments partially, though not fully, rewires the tissue-specific identity of macrophages, indicating a limited degree of plasticity even under such non-physiological conditions (70, 91).

At the same time, the activation state of terminally differentiated macrophages can vary as a function of microenvironmental signals, in particular cytokines. Historically, it has been thought that macrophages polarize into either classically activated, pro-inflammatory (“M1”) or alternatively activated, anti-inflammatory (“M2”) (92, 93) subtypes in response to cytokines associated respectively with type I or type II immunity (94, 95). However, it is now abundantly clear that this strict dichotomy is a drastic oversimplification of real-life *in vivo* physiology. Rather, these opposing polarization states represent extremes (94, 95) of a much wider and more fluid spectrum of activation states (96, 97). Understanding the different activation states of macrophages and monocytes in OA and the signals that drive them will be paramount in delineating their respective contribution to disease pathogenesis. Since circulating monocytes represent a modifiable source, they – and their relationship with macrophages found in the joints – are of particular translational relevance.

### Macrophage Niches

The intricate developmental dynamics between monocytes and macrophages and their adaptation to tissue-derived signals illustrate that these cell types actively engage with each other and their immediate environment or “niche”. Research into such niches represents a current focus in the field of myeloid cell biology. The niche concept postulates that macrophages are not only functionally imprinted by tissue-specific cues, but that their niches also provide them with a physical scaffold for anchoring and survival factors (17, 18). In turn, macrophages support appropriate functioning of their cellular partners. They thus form mutually beneficial cellular circuits (18) with their niches. In line with this, organ function is heavily impaired in mice lacking numerous tissue-resident macrophages owing to genetic deficiency in Colony Stimulating Factor (CSF)1, a key macrophage survival signal, or its receptor (98–100). Niches consist of macrophages and other, often non-hematopoietic stromal cell types, as well as the extracellular matrix surrounding them, and they can also “call” monocytes for replenishment. In the liver, for example, hepatocytes, endothelial and stellate cells together provide numerous signals to resident Kupffer cells and incoming monocytes, including CSF1, IL-34, CCL2 and Notch ligands (101), whereas in the red pulp of the spleen, macrophages depend on CSF1 produced by fibroblasts (102). In return, macrophages help facilitate tissue-specific functions and homeostasis. Beyond their role in immune surveillance and protective immunity, macrophages have been implicated in diverse physiological processes ranging from haemoglobin recycling, intestinal motility, surfactant degradation in the lung, to cardiac conduction (64, 102–109). The circuits underlying some of these less-traditional macrophage effects are starting to be deciphered. For example, macrophages located in the interstitial space of the testis produce cholesterol, which stimulates steroidogenesis in Leydig cells (110–112).

Whilst their cellular partners, signaling circuitry and functions remain incompletely understood, it is highly conceivable that distinct macrophage niches also exist in the joint. In the following, we will thus discuss how the current concepts of monocyte and macrophage biology in other tissues and organs reviewed above, apply to synovial joints, with particular emphasis on molecular and cellular mechanisms bearing potential for translational exploitation in OA. By interpreting the dynamics between these pleiotropic cell types and their functions within their potential joint-associated niches, we aim to provide an integrative view of their contribution to joint health and disease.

## Monocytes and Macrophages in Joint Homeostasis

### Bone and Adipose Tissue

The bone-resident macrophages are known as osteoclasts, peculiar large and multinucleated cells whose primary function is bone resorption. They are essential for skeleton remodeling and maintenance of the hematopoietic environment in the BM. Consequently, defects in osteoclasts cause osteopetrosis and hematopoietic failure, while their overactivation leads to osteoporosis. Osteoclasts allow for homeostatic bone turnover in joint-associated subchondral bone in response to loading. Osteoclasts first colonize the ossification centers of developing bones in the fetus from EMP, where they form long-lived syncytia that are maintained throughout life by low-grade fusion with incoming monocytes (113, 114). Adding to this complexity, elegant recent intra-vital imaging has shown that osteoclasts do not necessarily undergo apoptosis following activation and bone resorption, but instead, can fission into daughter cells termed “osteomorphs” (115). These can be recycled by fusion with osteoclasts but remain transcriptionally distinct from both osteoclasts and other macrophages.

As in many other tissues, adipose tissue-resident macrophages are developmentally and functionally heterogeneous. In the healthy adipose tissue of lean mice and likely humans (116, 117), monocyte-derived macrophages co-exist with long lived, fetal yolk sac EMP-derived macrophages and regulate appropriate development of adipose tissues and lipid storage during homeostasis (116, 118). Of note, it is currently unclear whether the macrophage compartments within joint-specific adipose tissues, such as the infra-patellar fat pad, are developmentally and functionally equivalent to those in more commonly studied adipose depots, such as the subcutaneous or inguinal fat. The infrapatellar fat is highly vascularized and innervated, and thus more reminiscent of visceral than subcutaneous fat (Reviewed in Urban and Little 2018) (30). It is also interesting to note that although generally considered a type of white adipose tissue, the infrapatellar fat may not always behave like other adipose tissues, for example in conditions of obesity. Although the infrapatellar fat pad increases in volume, vascularization and adipocyte size in response to obesity like other adipose tissues, it may be more protected from obesity-induced inflammation (119–121). This suggests that infrapatellar fat may show features of both white and brown adipose tissue in response to obesity, and distinct responses to other white adipose deposits have also been observed in OA (122). In end-stage knee OA patients the infrapatellar fat pad had significantly less macrophages, toll-like receptor 4 expression and fibrosis compared with other peri-synovial adipose tissue. In these same patients both adipose tissues had increases in adipocyte size and haematopoietic and M2 macrophage cell infiltration correlated with body mass index. This complex interplay between systemic and local joint factors related to post-traumatic OA, and how these affect and are affected by infrapatellar fat pad macrophage polarization, has been demonstrated in mouse models (123). The infrapatellar fat has been implicated as a major player in sustaining and perpetuating inflammation in OA (29). While macrophage deregulation has been associated with pathological changes in other adipose depots, those in the infrapatellar fat can contribute directly or indirectly to OA pathogenesis and future research is needed to better characterize which macrophage features it shares with other adipose tissues and which are unique.

### Synovial Membrane and Interstitial Connective Tissue

At homeostasis, macrophages are virtually the only immune cells in the synovial membrane (124, 125), and whilst the underlying interstitial connective tissue does harbor other lineages like mast cells and lymphocytes, macrophages predominate by far (126). Importantly, both the steady state synovial membrane and interstitium are largely devoid of monocytes. Healthy synovial tissue contains three populations of macrophages that are dynamically interconnected: lining macrophages gradually turn over from proliferating MHCII^+^ macrophages found in the sub-lining connective tissue, which also generate a second population of interstitial macrophages characterized by expression of Hypoxia-Induced Mitogenic Factor (Resistin-like alpha; RELMa) (22). The exact sources from which synovial macrophages are originally established during development remain to be determined with appropriate additional fate mapping systems. However, chimeras and parabiosis have now firmly established that all three populations receive minimal if any monocyte input in the adult steady state (22).

Despite their developmental interdependence, the distinct populations of synovial macrophages are phenotypically and functionally highly specialized. In addition to being a source of other synovial macrophages, MHCII^+^ sub-lining macrophages are particularly well-equipped for antigen presentation, while the RELMa^+^ population shows a regulatory phenotype and abundantly expresses scavenger receptors like CD206 and CD163 (22). Lining macrophages protect joint functionality and the immune privilege of the joint space through a multitude of mechanisms: they act as sentinels for molecular and cellular changes in the joint cavity (124) and facilitate clearance of cartilage and bone debris, highly immunogenic and hence dangerous signals that are constantly shed into the synovial fluid due to mechanical shear stress. In both mice and humans, lining macrophages express high levels of scavenger receptors, in particular Triggering Receptor Expressed on Myeloid Cells 2 (TREM2) and CD163 and are highly phagocytic and anti-inflammatory (22, 124, 127–129). Lining macrophages also actively participate in production of extracellular matrix (ECM) components and synovial fluid (22). Finally, sophisticated genetic and imaging approaches recently revealed that reminiscent of epithelial cells, lining macrophages form tight junctions with one another and thereby constitute a structural and immunological barrier (22). This barrier limits immune cell trafficking across the synovial membrane and thereby protects the avascular cavity from systemic threats. Conversely, it shelters the synovial connective tissue from immunogenic stimuli present in the joint space. Collectively, these features make synovial macrophages key regulators of joint homeostasis.

### Potential Macrophage Niches and Signals in Healthy Joints

**Figure 2:**
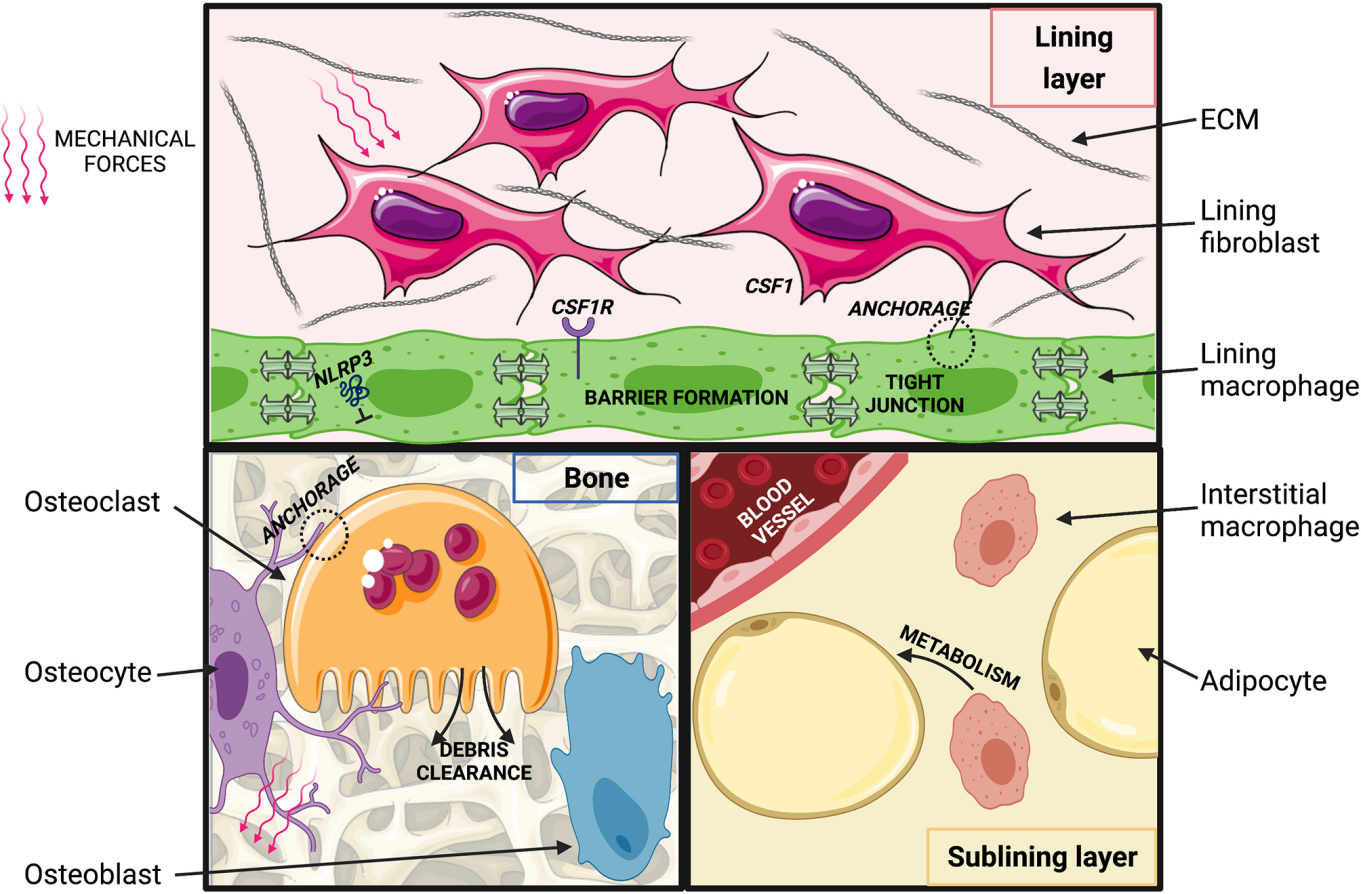
Putative macrophage niches in the healthy joint. (Top) Synovial lining: macrophages are connected *via* tight junctions and are in close contact with fibroblasts. Fibroblasts may provide CSF1 and anchorage to macrophages, which may be imprinted by exposure to ECM degradation products. Movement-induced cyclic stretch may inhibit NLRP3 inflammasome activation. (Bottom, left) Osteoblasts lining the bone surface synthesize bone matrix in response to soluble mediators released by osteocytes that sense changes in mechanical loading and bone deformation. Osteoclasts resorb bone and thereby regulate balanced homeostatic bone turnover (“modelling”) in response to anchorage and soluble signals from osteocytes and osteoblasts. (Bottom, right) In the synovial sub-lining, self-maintained interstitial macrophages may regulate adipose tissue metabolism and act as a reservoir to replenish synovial lining macrophages. Created with BioRender.com and smart.servier.com.

The exact cellular interactions and molecular signals comprising macrophage niches in healthy joint tissues remain to be deciphered with state-of-the-art approaches, however, fibroblasts are likely key players. This is the case for the spleen and peritoneal cavity (102, 130) and may also be particularly true for the synovial lining, where in the absence of a basement membrane they are in intimate contact with lining macrophages. Synovial fibroblasts and macrophages have been characterized individually in great detail over the last several years (22, 131, 132), and their potential interplay has been discussed in excellent recent reviews (133–135). Fibroblasts are ideally suited to provide anchorage to macrophages, and they are also a recognized source of key macrophage survival factors, such as CSF1 (**Figure 2**). Synovial lining macrophages are lacking in CSF1-deficient osteopetrotic (“op/op”) mice (99) demonstrating their CSF1-dependence, at least during development. Intriguingly, systemic administration of CSF1 does not restore synovial macrophages, whereas transgenic overexpression of the full-length transmembrane protein does, suggesting they depend on the membrane-bound isoform of the growth factor and thus, local sources (99, 136). Whilst synovial lining macrophages express the receptor for CSF1 receptor (CSF1R) at steady state (22), it is currently unclear whether they also rely on CSF1 for their homeostatic maintenance and turnover.

Fibroblasts could also act to bring macrophages in proximity of tissue-specific cues that imprint their functional identity, although this process may be orchestrated by additional stromal cell types in the joint, such as adipocytes, endothelial cells and chondrocytes. Such complexity is seen in the liver, where incoming monocytes are functionally imprinted by a triad of hepatocytes, endothelial cells and stellate cells (101). Joint tissues are constantly exposed to shear stress and tensile forces that dynamically change with variations in joint loading. Mechanical stimuli are amongst the candidate cues that could play a particularly important role in instructing the specific identity of macrophages in joint tissues. Macrophages are in principle capable of sensing mechanical forces. Human BM-derived macrophages for example respond to substrates with different stiffness with changes in their shape, migration and proliferation (137). Mechanotransduction can also directly modulate their inflammatory cytokine production (138, 139). This latter effect is dependent on the NLRP3 (NOD-, LRR- and pyrin domain containing 3) inflammasome and can also involve signaling through TRPV1 and 4 (Transient receptor potential vanilloid-type 1 and 4) cation channels, which have been implicated in ptOA pathophysiology in mice (140–142). Transduction of mechanical signals through TRPV4 has also been implicated in the formation of multinucleated giant cells, inflammatory and destructive multinucleated macrophages (143). In addition to mechanical stress, normal ECM turnover products represent candidate signals that could imprint joint macrophage identities. Indeed, synovial lining macrophages appear to be highly phagocytic and constantly scavenge cartilage debris from the joint cavity (144–146). Joint biomechanics are altered during OA pathogenesis, and joint-tissue ECM degradation products more prevalent than at homeostasis, thus these pathways likely also impact macrophage identities and functions in arthritic joints.

## Monocyte and Macrophage Functions in the Injured and Osteo-Arthritic Joint

### Macrophage Functions in ptOA Pathogenesis

In addition to self-maintained lining and interstitial sub-lining macrophages already present at steady state, the arthritic synovium contains inflammatory monocyte-derived macrophages (147, 148). Similar changes also occur in other tissues within the joint (e.g. subchondral bone), regional (e.g. lymph node) and in remote tissues (e.g. spleen, peripheral blood). The necessity to delineate the specific roles of these distinct macrophage populations is highlighted by discrepant findings on the consequences of macrophage depletion depending on the experimental approach. Although the precise contribution of the various populations remains to be shown, systemic depletion of macrophages in mice in which apoptosis is induced in cells expressing CSF1R exacerbates experimental ptOA, whereas local clodronate liposome-mediate depletion within the joint is beneficial (149).

In RA, the respective functions of the distinct macrophage subsets have now been well explored, and macrophages originating from recruited monocytes appear to have overall disease-promoting functions (150, 151). Similarly, the majority of studies on OA-affected joints have identified inflammatory, monocyte-derived macrophages as the main culprit in promoting and sustaining inflammation (124, 152). These cells produce pro-inflammatory cytokines and release additional signaling molecules associated with tissue-injury, which can attract lymphocytes that further propagate inflammation. However, exploiting these findings therapeutically is currently hindered by a lack of detailed understanding of the exact interplay between monocyte-derived macrophages and different types of lymphocytes, and how these change in the distinct stages of ptOA pathogenesis. Monocyte-derived macrophages also participate in cartilage destruction *via* production of IL-1β and TNF-α, which suppress synthesis of the ECM components aggrecan and collagen by chondrocytes and upregulate expression of catabolic enzymes like ADAMTS-4 and MMP-13 (153–155). Soluble matrix degradation products in turn can activate resident synovial macrophages *via* Toll-Like Receptors (TLRs) and other pattern-recognition receptors (156). As this example illustrates, different macrophage populations in the joint can be functionally interlinked.

Another effector by which macrophages might contribute to ptOA pathogenesis is B cell Activating Factor (BAFF), a member of the TNF superfamily. BAFF is a crucial B cell survival factor, but also exerts co-stimulatory effects on T cell activation *via* upregulation of B-cell lymphoma 2 (BCL-2) (157). Furthermore, BAFF promotes T-helper-cell (Th)1 and suppresses Th2 responses (158), and drives Th17 differentiation *via* Il-6 signaling (87, 158–161). BAFF levels are elevated in serum and synovial fluid from RA patients (162), and BAFF appears to have a pathogenic role in RA (163, 164). During established RA, BAFF promotes pro-inflammatory polarization of CD4^+^ T cells, DC maturation as well as proliferation of inflammatory fibroblasts (163). In the inflamed joint, macrophages (165) are the main source of BAFF, although it is unclear if these are monocyte-derived or resident macrophages, or both. This compelling evidence led to the development of BAFF antagonists as DMDs for RA, which are currently being tested in early phase clinical trials (163). Whether BAFF production is also a mechanism by which macrophages contribute to pathogenesis of OA has not been determined but elevated BAFF levels have been detected in OA synovial fluid (166).

Unlike their monocyte-derived counterparts, and some controversy notwithstanding, self-maintained resident synovial macrophages have largely been attributed protective roles in arthritis. The barrier generated by synovial lining macrophages is disrupted in both RA patients and experimental RA (22). In mice, this occurs rapidly upon induction of serum transfer-mediated arthritis, and thus constitutes an early event in disease development. In this model, barrier breakdown occurs following phagocytosis of immune complexes containing auto-antibodies, which activate lining macrophages and induce structural joint pathologic changes. Consequently, depletion of lining macrophages or specific disruption of their tight junctions worsens experimental RA. In turn, drug-mediated stabilization of tight junctions protects mice from RA (22), a finding that is translationally promising. Whilst the role of synovial lining macrophages has not yet been addressed specifically in OA pathogenesis, it is worthwhile noting that targeting lining macrophages or tight junctions not only exacerbates RA but may also result in spontaneous inflammation in the joint cavity in otherwise healthy animals (20, 22). With respect to ptOA, one could thus envision a scenario in which following injury, mechanical disruption of the synovial lining macrophage barrier enables rapid influx of inflammatory cells and hence, transition to the inflammatory phase of OA pathogenesis. Unlike in RA however, this barrier breach might be transient in nature, since the barrier appears more intact in patients with established OA compared to RA (22). This might be due to differences between immune complex-mediated and mechanical barrier-breakdown and could contribute to the, often considerable, lag phase between joint injury and ptOA onset.

### Monocyte Functions in Joint Pathogenesis

Monocytes are critical players in OA pathogenesis, both as effector cells and a source of additional macrophages. As described earlier, it is widely accepted that Ly6C^high^ and Ly6C^low^ monocytes can differentiate into macrophages with distinct polarization profiles in response to the cytokine milieu encountered in the tissue microenvironment. Reflecting this complexity, the overall impact of classical and non-classical monocytes on joint disease pathogenesis remains unclear. On the one hand, adoptive transfer of Ly6C^low^ monocytes following pan monocyte depletion increases the development of serum-transfer induced arthritis (58, 167, 168). In this model, Ly6C^low^ monocytes are actively recruited to the joint, where they are critical for the initiation of sterile joint inflammation and differentiate into inflammatory macrophages (169). On the other hand, Ly6C^low^ monocytes were also found to limit excessive inflammation in arthritic mice *via* enhanced recruitment of regulatory T-cells (Tregs) (58, 170). This seemingly contradictory evidence regarding the role of Ly6C^low^ monocytes in RA underscores the need for further studies that improve the understanding of the complex role of monocytes in inflammatory arthritis, and similar considerations apply to OA. The diverse roles of monocytes and macrophages in ptOA pathogenesis will be discussed in more detail in the following section, focusing on molecular and cellular factors shaping their respective functions.

## Signals and Cellular Interactions Shaping Monocytes and Macrophages in ptOA

**Figure 3:**
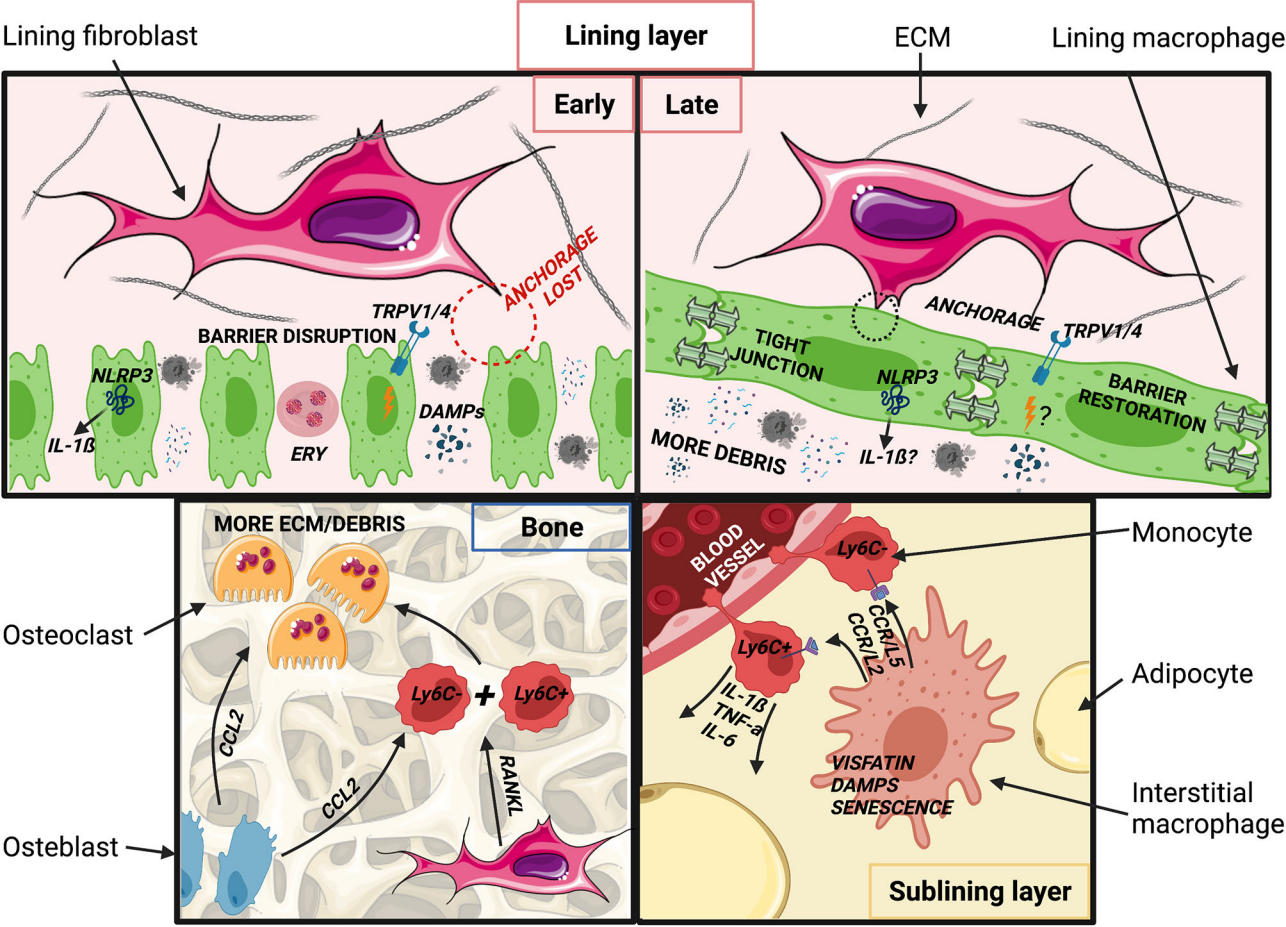
Putative changes in joint tissues after injury and during post-traumatic OA development. (Top, left) Acutely following injury, synovial lining macrophages are spatially re-orientated and the barrier is disrupted. DAMPs, PAMPs and catabolic enzymes are released into the synovial cavity by chondrocytes and damaged tissues (ligament, meniscus). Extra-vascular erythrocytes and associated free heme from blood vessel injury may pathologically imprint synovial macrophages. Barrier disruption may impede cyclic stretching of lining macrophages, resulting in NLRP3 inflammasome activation and increased IL-1ß production, known to promote of OA. Altered mechanics may also promote joint inflammation through TRPV1/4 cation channels. (Top right) At later stages of ptOA pathogenesis, the synovial lining layer may be restored. Levels of IL-1ß remain elevated, though involvement of the NLRP3 inflammasome is unclear. TRPV1 activation may continue to promote OA pathogenesis, although likely *via* signals other than or in addition to mechanical stimuli. Cellular debris, DAMPs and PAMPs remain abundant in the synovial cavity and thus potentially imprint pathological macrophage phenotypes. (Bottom, left) Increased numbers and activation of osteoclasts contribute to accelerated bone turnover and remodeling in the arthritic joint. Osteoclastogenesis may be promoted by CCL2 produced by activated osteoblasts and inflammatory cells, potentially resulting in recruitment and fusion of Ly6C^high^ and Ly6C^low^ monocytes, a process that may be further stimulated by RANKL produced by lining fibroblasts. (Bottom, right) In the sublining layer, exposure to ECM degradation products may stimulate interstitial macrophages to produce CCL2 and CCL5, leading to recruitment of Ly6C^high^ and Ly6C^low^ monocytes. Ly6C^high^ monocytes produce IL-1ß, TNF-α and IL-6, potentially in response to the adipokine visfatin, a TLR4 receptor agonist, which also induces changes in the subchondral bone. Ly6C^low^ monocytes may supply the interstitial macrophage pool, but these macrophages may retain higher baseline NF-κB and IL-1ß activity than those in healthy joints. Created with BioRender.com and smart.servier.com.

Depending on severity, joint injury can induce marked mechanical, anatomical and immunological changes, initially resulting in recruitment of monocytes and other inflammatory cells. Pathological changes persist throughout ptOA development and in established disease, and impact both incoming monocytes and previously resident macrophages. This section discusses how the perturbed joint tissue environment might affect monocytes and macrophages (**Figure 3**). An overview of murine and human monocytes and macrophages found in the synovial tissue during homeostasis, rheumatoid arthritis and in as far as known osteoarthritis, can be found in **Table 1**.

**Table 1:** Markers, origin and putative function of monocytes and synovial macrophage subsets in mouse and human.

Population, location	Phenotype	Origin, maintenance	Functions at homeostasis	Functions in osteoarthritis
	Mouse: TREM2^+^ CX3CR1^high^ MHCII^-^	Long-lived, BM-independent, not proliferating^2^,^3^ replenished from sublining interstitial	Protective barrier^2^	Protective barrier,^2^ limiting disease development (RA), immune regulation^3^
Macrophages, synovial lining	“mTOR activated M1 macrophages” iNOS^+1^	Unknown	Unknown (low number)	Chondrocyte differentiation^1^
	Human: TREM2^+^ CD68^+^ MerTK^+^ LYVE1^+^ FOLR2^+4,2^	Proliferation^4,5^	Protective barrier^2^ Control local immune responses^4^	Protective barrier^2^ Resolution of inflammation, induce reparative fibroblast
	CD14^+^HLADR^+^ FOLR2^+^CD86^+5^ KI67^high^ ULK1^+^	Proliferation^5^	Unknown	
	CD14^+^HLADR^+^ FOLR2^+^CD86^+^ KI67^low^ *HTRA1+* (location uncertain)	Uncertain	Unknown	Cartilage remodelling^5^
Macrophages, synovial sublining/ interstitital	Mouse: RELMa^+^ CD206^+^ CD163^+^	BM-independent^2^	Joint homeostasis	
	CX3CR1^−^ MHCII^+^ CSF1R^+^	Proliferation, BM-independent (RA)^2^ / BM (arthritis)^3^	Generate lining and sublining macrophages^2^	Generate lining and sublining macrophages^2^, Inflammation
	Human TREM2^-^ MerTK^-^ CD206^-^ CLEC10a^+42^	Unknown	Unknown	Fibroblast inflammatory response^4^
Macrophages, synovial tissue of the “bare area”	Mouse: CX3CR1^+^ MHCII^+^ Ly6C^int^F4/80^+^	BM^6^	Absent	Osteoclastogenesis^6^
	Human: CX_3_CR1^+^ HLA-DR^high^ CD11c^+^ CD80^-^ CD86^+^	Unknown	Unknown	Osteoclastogenesis^6^
Monocytes, synovial tissue	Mouse: Ly6C^+^ CD64^int^	BM^3^	Tissue patrolling	Generate Ly6C^-^ monocytes
	Ly6C^-^	BM^3^	Absent	Initiation of sterile joint inflammation^3^ Generate MHCII^+^ macrophages^3^
	Human: CD14^+^ CD11c^+^ CD38^+^ IL1B^+^ IFN-activated SPP1+^7^		Unknown	Pro-inflammatory^7^
	CD14^+^ CD11c^−7^ *NUPR1^+^* ^7^		Joint homeostasis^7^	Inversely correlated with tissue inflammation, bone remodelling^7^

TREM2, triggering receptor expressed on myeloid cells 2; CXCR1, C-X3-C Motif Chemokine Receptor 1; MHCII, major histocompatibility complex class II; mTOR, mammalian target of rapamycin; iNOS, inducible nitric oxide synthase; BM, bone marrow; RA, rheumatoid arthritis; MerTK, MER Proto-Oncogene, Tyrosine Kinase; LYVE1, Lymphatic Vessel Endothelial Hyaluronan Receptor 1; FOLR2, Folate Receptor Beta; CD, cluster of differentiation; HLADR, Human Leukocyte Antigen – DR isotype; ULK1, Unc-51 Like Autophagy Activating Kinase 1; HTRA1, HtrA Serine Peptidase 1; RELMa, Resistin-like molecule a; CSF1R, colony-stimulating factor 1 receptor; CLEC10a, C-type lectin domain family 10; Ly6, lymphocyte antigen 6; IL, interleukin; IFN, interferon; SPP1, Secreted phosphoprotein 1; NUPR1, Nuclear Protein 1.

^1^Zhang, H. et al. Synovial macrophage M1 polarisation exacerbates experimental osteoarthritis partially through R-spondin-2. Ann. Rheum. Dis. 77, 1524–1534 (2018).

^2^Culemann, S. et al. Locally renewing resident synovial macrophages provide a protective barrier for the joint. Nature 572, 670–675 (2019).

^3^Misharin, A. V. et al. Nonclassical Ly6C- monocytes drive the development of inflammatory arthritis in mice. Cell Rep. 9, 591–604 (2014).

^4^Alivernini, S. et al. Distinct synovial tissue macrophage subsets regulate inflammation and remission in rheumatoid arthritis. Nat. Med. 26, 1295–1306 (2020).

^5^Wood, M. J. et al. Macrophage proliferation distinguishes 2 subgroups of knee osteoarthritis patients. JCI Insight 4, (2019).

^6^Hasegawa, T. et al. Identification of a novel arthritis-associated osteoclast precursor macrophage regulated by FoxM1. Nat. Immunol. 20, 1631–1643 (2019).

^7^Zhang, F. et al. Defining inflammatory cell states in rheumatoid arthritis joint synovial tissues by integrating single-cell transcriptomics and mass cytometry. Nat. Immunol. 20, 928–942 (2019).

### Perturbations Following Joint Injury

Joint injury triggers a series of complex mechano-biological and immunological changes, which can be broadly separated into three successive phases. Immediately after injury, mechanical perturbation effects predominate. These are direct results of the injury (9, 171) and may include tissue disruption (e.g., subchondral bone (micro)fractures, ligament tearing), collagenous matrix disruption and cartilage swelling, blood-vessel injury and hemarthrosis i.e. the presence of blood in the synovial cavity. This immediate joint-tissue injury is followed by an acute inflammatory phase (172), which is characterized by abundant cell death and pro-inflammatory signaling involving both innate and adaptive lineages (173). The nature and duration of this inflammatory response have been identified as major determinants for the risk of developing ptOA post injury (9, 172, 173). While appropriate control and resolution of inflammation is essential for normal wound-healing and might prevent ptOA development, perpetuated inflammation leads to the chronic and final phase of OA pathogenesis, which is defined by fluctuating low-level synovitis (174) and continuous tissue remodeling processes that ultimately lead to destruction of the cartilage and joint failure (175). Delayed or failed resolution of inflammation can be due to a permanently disrupted equilibrium of pro- and anti-inflammatory factors (171) or inadequate post-injury inflammation control (172). In addition, biomechanical factors such as instability or recurring joint injuries (176) can result in continuous re-triggering of acute mechano-biological responses, which initiate an inflammatory vicious circle. These considerations identify the acute inflammatory phase as a potential target for ptOA DMDs and highlight the need for a better understanding of its cellular and molecular regulators.

### Mechanisms Underlying Monocyte Recruitment During OA Pathogenesis

The exact nature of the inflammatory response subsequent to joint injury is still under investigation, and differences might exist depending on the type of injury and/or tissues injured, as we will discuss below. Overall, however, a growing body of evidence implicates chemokines and their receptors in monocyte recruitment during OA pathogenesis. As reported for other pathologies (177), classical and non-classical monocytes differentially depend on CCL2/CCR2 or CCL5/CCR5 and CX3CL1/CX3CR1. CCL2 (also known as MCP-1) is a key regulator of Ly6C^high^ monocyte egress from the BM and their recruitment to peripheral tissues (178). Following joint injury, release of ECM degradation products and complement factors temporally induces CCL2 production by chondrocytes, resident synovial macrophages (179) and endothelial cells (180). This occurs *via* a positive feedback loop, stimulated by increased IL-1β and TNF-α expression in synovial macrophages (181) and fibroblasts (182). In keeping with this, clinical studies have reported elevated levels of CCL2 in synovial fluid immediately after traumatic joint injury (183) and subsequent to meniscal tears (184), and concentrations correlate with severity of OA (185, 186). Its expression is also significantly elevated in the serum of OA patients (187), and CCL2 might also affect other cells relevant to OA pathogenesis. In chondrocytes, for example, CCL2 increases expression of the catabolic enzymes MMP3 and MMP13 (179) and inhibits proliferation and enhances apoptosis. CCL2 might thus promote OA pathogenesis *via* attracting monocytes to the joint, but also by directly promoting cartilage destruction (179).

Unlike CCL2, which is found mainly in the intimal lining of the synovium, CCL5 [also known as RANTES (Regulated on Activation, Normal T Cell Expressed and Secreted)] is distributed more diffusely throughout the synovial tissue (188). Conflicting evidence exists regarding the role of CCL5 and its receptor CCR5, which possess strong chemo-attractive properties for Ly6C^low^ monocytes. In line with a disease-promoting role of Ly6C^low^ monocytes, CCR5^-/-^ mice were initially reported to exhibit reduced cartilage destruction (189), however, a recent study by Raghu and colleagues found that neither deficiency in CCL5, nor CCR5 protects mice from ptOA (21). This was further corroborated by a clinical study of synovial biopsy specimens, which found significantly higher levels of CCL5 in RA compared to OA patients, whilst expression of all other chemokines and receptors is comparable (188). This may suggest that the inflammatory responses underlying these different arthritides have some unique molecular signatures or phenotypes. Considering its role in attracting Ly6C^low^ monocytes, which are known to promote pathogenesis of inflammatory arthritis, it seems surprising that depletion of CCR5 has no protective function in ptOA (21). However, CX3CL1/CX3CR1 signaling also participates in recruitment of non-classical monocytes, and elevated levels of CX3CL1 have been found in peripheral blood (190) and synovial fluid (186) of OA patients. Functional experiments revealed that in addition to its chemo-attractive properties, CX3CL1 also stimulates inflammation specifically at the early stages of OA (190). Finally, soluble CX3CL1 induces production of the pro-inflammatory cytokines IL-1β, IL-6 and TNF-α in recently recruited monocytes (191). Taken together these data indicate that the CX3CL1/CX3CR1 axis predominates in recruitment and pro-inflammatory activation of Ly6C^low^ monocytes in the context of OA initiation. Ly6C^high^ monocytes recruited and activated *via* CCL2/CCR2, on the other hand, might help sustain inflammation at later stages (192). Temporal changes in chemokine expression and associated monocyte sub-population recruitment and accumulation, may in part explain the recently described loss with time post-injury, of an initially protective/anabolic effect of injured synovium on chondrocytes (193).

Osteoclasts have been implicated in progressive joint destruction. Osteoclastogenesis is controlled by RANK (Receptor Activator of Nuclear factor Kappa B (NF-κB) (194). Its ligand (RANKL) is expressed by fibroblast-like synoviocytes and Th17 cells, and expression is regulated by pro-inflammatory cytokines secreted by monocytes and macrophages (IL-1β, IL-6, IL-17 and TNF-α) (195). A particularly interesting mechanism by which monocytes could contribute to increased osteoclastogenesis in the context of arthritis was proposed by Hirose *et al.* They postulated that CCL2 secreted by osteoblasts leads to fusion of Ly6C^high^ with Ly6C^low^ monocytes stimulated by RANKL, resulting in mature, multinucleated osteoclasts (195). In addition to osteoblasts, activated inflammatory cells produce large amounts of CCL2 and this might explain the accelerated osteoclastogenesis and joint destruction in an inflammatory setting (195).

### Signals Governing Monocyte Differentiation, Activation, and Functions in OA

Following their recruitment to the tissue, monocyte functions can be shaped by a variety of signals present in the non-homeostatic joint. Calcium binding proteins can act as damage- or pathogen-associated molecular patterns (DAMPs and PAMPs, respectively) and have multi-faceted effects on OA pathogenesis. Damage to the cartilage leads to increased levels of S100A8 and its binding partner S100A9 specifically in synovial pro-inflammatory macrophages, but not fibroblasts (196). Secretion of these factors induces the production of pro-inflammatory cytokines in these macrophages in an autocrine manner (196). In addition, in a murine collagenase-induced OA model, which has a more inflammatory phenotype than surgically-induced disease, release of S100A8/9 elicits influx of Ly6C^high^ monocytes *via* upregulation of CCL2 (197) and increased egress of Ly6C^high^ monocytes from the BM (197). In turn, activated monocytes are a major source for S100A8/9, which might thus constitute a positive feedback loop. Finally, S100A8/9 might also be derived from chondrocytes and directly contribute to cartilage disruption in OA by inducing production of ADAMTS-4 and -5, MMP-1, -3, -9 and -13 and the pro-inflammatory cytokines IL-6, IL-8 and CCL2 in chondrocytes in a TLR4-dependent manner (198, 199). Interestingly, chondrocyte-derived S100A8/9 may play a predominant role in the acute post-injury phase, as expression and protein levels in cartilage decrease with post-traumatic OA disease progression while levels are maintained in immune-mediate inflammatory arthropathy (199). S100A8/9 thus fuel the initial pro-inflammatory microenvironment in the joint, provide chemotactic cues for Ly6C^high^ monocytes and exert direct catabolic effects within the cartilage, processes which are further amplified by several feedback loops. Another class of OA-associated DAMPs are basic calcium phosphate (BCP) crystals. Whilst *in vivo* data on their relevance to OA are currently lacking, *in vitro* exposure of monocyte-derived macrophages to BCP crystals leads to a classically activated, pro-inflammatory phenotype, a bioenergetic switch towards glycolysis and increased expression of S100A8 (200). Promisingly, both BCP-induced phenotypic polarization and S100A8 expression are inhibited by a glycolytic inhibitor (2DG) indicating that metabolic reprogramming might be underlying these effects (200).

The Janus Kinase/Signal Transducer and Activator of Transcription (JAK/STAT), Mitogen-Activated Protein Kinase (MAPK) and NF-κB pathways are involved in differentiation of monocytes into macrophages and their functional polarization. The latter depends on interferon regulatory factors (IRFs) (201). IRF5 is a also downstream target of Granulocyte-Macrophage Colony-Stimulating Factor Receptor (GM-CSFR), and plays a critical role in pro-inflammatory macrophage polarization (202). A recent clinical study investigated the role of IRF5 in OA and found it to be overexpressed in synovial macrophages, but not circulating monocytes (203). However, exposure to synovial fluid from OA patients induced expression of IRF5 and IL-12 (via the individual subunits IL-12p35 and IL-12p40) in monocytes, in turn making them potent inducers of a Th1 response characterized by expression of IFN-γ and Tbx21 in co-cultured naïve CD4^+^ T-cells (203). This suggests that patient synovial fluid contains soluble factors capable of inducing IRF5 in monocytes, thus contributing towards a Th1 inflammatory response.

TLR 4 is a receptor for PAMPs and DAMPs expressed on monocytes that plays an important role in the activation of innate immunity (204), and has been implicated in the inflammatory reaction associated with OA (205). The adipokine visfatin was recently identified as a TLR4 receptor agonist capable of evoking inflammatory responses (206). In addition, visfatin stimulates production of IL-1β, TNF-α and IL-6 by monocytes (207), and the resulting inflammatory environment displays higher levels of circulating visfatin, thus constituting a positive feedback loop (208, 209). Visfatin is also involved in inter-tissue joint communication underlying changes in the subchondral bone (210). These have long been described in OA, but the exact mechanisms of this remodeling and pathways of its activation has remained elusive. Emerging evidence now points towards direct communication between the subchondral bone and cartilage *via* diffusion (211). Laiguillon *et al.* found that visfatin is produced in cartilage, synovium and subchondral bone and exerts an enzymatic effector function selectively inducing a pro-inflammatory phenotype in chondrocytes, osteoblasts and synoviocytes, characterized by increased secretion of IL-6 and CCL2 (210, 212–214). Because of its contribution to the inflammatory response and tissue remodeling, inhibition of visfatin might be a promising DMD approach.

The gene expression profiles of monocytes and monocyte-derived macrophages, and hence, their functional polarization, might also be shaped by microRNAs expressed in response to environmental stimuli. A recent study identified miR-155 as a potential genomic switch in monocyte-derived macrophages generated *in vitro*, which regulates their inflammatory profile (215). Intriguingly, this phenomenon is partially reversed by treatment with monoclonal anti-TNF antibodies, but not a soluble TNF receptor (Etanercept) (215). MiRNAs expressed by monocytes and their macrophage progeny, such as miR-155, might therefore represent promising candidate DMD targets.

While we have focused primarily on joint injury and ptOA, monocytes and macrophages in the arthritic joint might also be affected by age and cellular senescence, as has been demonstrated for RA. In this context, an elegant mouse study from Misharin *et al.* is of note, where the role of different monocyte subsets in RA pathogenesis using serum-transfer induced arthritis was investigated. Ly6C^low^ monocytes are recruited to the joint and initially develop into classically activated macrophages, but the macrophage compartment gradually undergoes a switch towards a more alternatively activated phenotype (169). The initial highly pro-inflammatory nature of the Ly6C^low^ monocytes could be caused by a senescence-associated secretory phenotype, which is associated with high baseline NF-κB and IL-1α activity (216). In addition, accumulation of Ly6C^low^ monocytes is found in the elderly. These findings suggest that senescence might correlate with increased numbers and pro-inflammatory skewing of Ly6C^low^ monocytes, which might further exaggerate the inflammatory response unfolding during RA progression. Whether similar mechanisms might be at play in ptOA requires evaluation, but it is interesting to note the accelerated disease progression, increased expression of inflammatory genes and inhibitory effects of MIF ablation following medial meniscal destabilization in older versus younger mice (217, 218).

### Immunogenic and Imprinting Signals in the Injured Joint

As introduced, the synovial membrane plays a key role in maintaining joint homeostasis as it guarantees the relative immune privilege of the synovial cavity. However, while the joint space itself is not vascularized, the synovial membrane also features a vascular net located just below the intima. This comprises capillaries, venules, arterioles and lymphatics (219) through which systemic and local inflammatory stimuli can be sensed (125, 220). Hemarthrosis and cartilage damage are direct consequences of joint trauma, which affect the joint not only macroscopically, but also on a cellular and molecular level (176). Of note, the presence of blood in synovial fluid is an independent predictor or poorer 2-year outcome following joint injury (221). It is tempting to speculate that heme could be a cellular cue shaping macrophages in the early stages of ptOA pathogenesis, but unlike in the homeostatic spleen, it may instruct more inflammatory cell states (222). Moreover, hemarthrosis directly activates the complement system, leading to production of complement anaphylatoxins (C3a and C5a) and formation of the membrane attack complex (223). Intriguingly, genetic deficiency for individual components of the complement system in mice leads to either attenuated [C5 and C6 (224)] or aggravated [CD59, also known as protectin (224)] ptOA joint damage following injury.

As discussed above, mechanical forces may directly shape macrophage functions in the homeostatic joint. It is therefore plausible that the mechanical changes following joint injury, impact on resident and recruited macrophages. This appears to be the case at least in experimental RA, where the extent of mechanical loading determines the local distribution of inflammation and degree of damage (225). Mechanical damage to the cartilage also leads to substantial ECM degradation. Collagen fibers fail to contract (226, 227) and ECM degradation is further enhanced by the lack of maintenance and repair (228) following chondrocyte death. ECM-derived tissue fragments are widely recognized as pro-inflammatory and immunogenic (229, 230). These fragments and the complement anaphylatoxin C5a can act as effective chemo-attractants for innate and adaptive immune cells (231, 232) and directly activate macrophages *via* NF-kB signaling (233). Released cartilage destruction products such as matrilin-3 (229, 234), tenascin-C (235), fragmented biglycan (236) and fibronectin (237) can also potently activate resident synovial macrophages. Finally, cartilage and other joint tissue degradation can induce release of additional DAMPs (238) capable of activating innate immune cells *via* TLR2 and 4 (239) and NF-kB signaling. The mechano-biological damage induced by injury thus generates an inflammatory microenvironment in the joint space, which is characterized by an increase in soluble inflammatory mediators and chemo-attractants that might induce transition to the acute inflammatory phase, and importantly shape subsequent responses of both recruited monocytes and resident macrophages.

### T Cell-Mediated Monocyte and Macrophage Activation in OA?

Other immune cells may provide signals amplifying the effects of monocytes and macrophages. In the context of joint injury, recruitment and activation of lymphocytes have traditionally been thought of as secondary events that follow monocyte influx and changes in macrophages (11). However, T cells are found in synovium at higher levels in early versus late OA (240) and might actively contribute to monocyte and macrophage activation *via* co-stimulatory pathways. One such pathway relies on interactions between CD40, a member of the TNF receptor family found primarily on antigen-presenting cells and monocytes, and its ligand CD40L (CD154), which is almost exclusively expressed by activated CD4^+^ T cells (241, 242). CD40/CD40L interactions elicit a broad pro-inflammatory response (243) that involves B cell differentiation (244) and macrophage activation. In turn, activated macrophages and other antigen-presenting cells enhance immunoglobulin antigen affinity (241), activate cytotoxic T cells and promote a Th1 immune response (245). This co-stimulatory pathway therefore has the potential to initiate a powerful amplification loop that propagates joint inflammation. In keeping with this notion, exaggerated CD40/CD40L signaling contributes to autoimmunity (246), including RA. Multiple studies have shown overexpression of both CD40L (247–249) and CD40 (246) in RA, and levels of CD40L are associated with disease activity (248) and perpetuation (247). Based on these findings, biological treatments targeting this axis in RA have been developed, which are currently undergoing early phase clinical trials (250). Despite differences in the pathogenesis and mechanisms involved in the development of RA and OA, shared elements in the underlying inflammatory response seem plausible (251). The effects of targeting the CD40/CD40L axis in OA remain to be determined, however we have found that CD40L mRNA levels are elevated exclusively in the synovium immediately after ACL rupture and during early onset of OA development (252). These preliminary findings support the notion that CD40/CD40L may be an early driver of T cell-mediated synovial macrophage activation and warrant future research into the CD40/CD40L axis specifically in ptOA.

### Additional Candidate Pathways and Mechanisms Leading to Macrophage Dysregulation During OA Pathogenesis

In addition to the factors discussed above, obesity is a well-established risk factor contributing to OA development (253), through increased mechanical loading but also *via* dysregulated secretion of adipokines and other metabolic factors (254). In mice, high-fat diet (HFD) results in elevated leptin-induced levels of lysophosphatidylcholine (lysoPC), which in turn increases MMP13 production by chondrocytes. As a consequence, obese mice show an earlier onset and progressive course of spontaneous OA (254). Direct links between obesity and OA have also been shown in mouse models of ptOA. HFD was associated with inflammation in the infrapatellar fat pad, characterized by macrophage crown-like structures, which may have a priming effect on the fat pad leading to a metabolic state of progressive OA following injury (123). HFD was also shown to aggravate inflammation of the synovial membrane post-injury, which was marked by increased macrophage infiltration (255). These findings are in line with the notion that obesity contributes to aberrant macrophage activation in OA pathogenesis. Intriguingly, these detrimental effects of HFD on OA persisted even after a normal diet was resumed (254), indicating long-lasting effects and potential windows or particular susceptibility. Some of these effects may even be programmed in early life and transmitted across generations. Indeed, increased higher susceptibility to experimental ptOA has been reported in the first and second generation offspring of mice fed a HFD during breeding (256). Immune cells have been implicated as mediators of such programming and transgenerational effects of obesity in offspring (256), and epigenetic dysregulation has been postulated as a central mechanism.

While some epigenetic modifications are stable and passed on across generations, others are more dynamic and responsive to environmental stimuli (257). These are believed to play a significant role in OA development. Of the studies that have investigated epigenetic changes in OA development, most have focused on epigenetic mechanisms modulating chondrocyte biology and inflammatory mediators (258). Evidence for epigenetic modifications of macrophage remains scarce in the context of OA. In principle, epigenetic processes govern various aspects of macrophage biology, including their development, differentiation, and activation, as well as the specification of their tissue identity (259–261). For example, active DNA demethylation occurs during monocyte to macrophage differentiation *in vitro* (262) and the identity of tissue-resident macrophages is shaped by unique enhancer landscapes in response to microenvironmental cues (70, 71). They also activate genes governing embryonic stem cell-like self-renewal through macrophage-specific enhancers (263). Fully differentiated macrophages are maintained in a “balanced” state through a combination of activating (such as PU.1, H3K4me1 and open chromatin) (264) and repressive (such as H3K9me3, H3K27me3 and H4K20me3) (265) epigenetic marks and regulators (262). These repressive marks are removed upon stimulation of macrophages through TLR, and specifically TLR4, ultimately resulting in the production of inflammatory cytokines such as IL-1β, CXCL10, IL-6 and TNF (262). TLR4 signaling has also been implicated in low-grade inflammation mediated by plasma proteins present in the synovial fluid of OA patients (266). Whether epigenetic changes in macrophages contribute to this remains to be formally shown, however.

Activation *via* TLR4 also initiates metabolic reprogramming of macrophages, and distinct metabolic states have been linked to functional differences in macrophage subsets. For example, metabolic reprogramming towards increased glycolysis promotes pro-inflammatory polarization (267). In OA, increased glucose uptake correlates with disease progression, and the hypoxic environment in the OA synovium may enhance osteoclastogenesis (267, 268). Osteoclastogenesis appears to also be promoted by metabolic syndrome through NF-κB activation and advanced glycation end products (269). A bioenergetic switch towards glycolysis is also induced in macrophages by basic calcium phosphate crystals, which are specifically found in OA (220), further supporting the notion that macrophages may undergo metabolic reprogramming during OA pathogenesis.

Finally, epigenetic and immunometabolic changes are also hallmarks of “trained immunity”. This recently coined concept (270) acknowledges that innate immune cells, including macrophages, show increased responsiveness to secondary stimuli following “training” by primary exposures. Whilst trained immunity has not specifically been studied in the context of OA, many of the cellular signals that impact on macrophages during OA pathogenesis – or even preceding disease onset - could mediate long-term effects through inducing this type of innate memory in macrophages. Thus, obesity/HFD may be primary exposures that train heightened or specific OA inducing immune responses to a secondary stimulus such as injury (123, 256). It is interesting to speculate that this may also be relevant in the context of prior even minor joint injuries increasing the risk and/or severity of ptOA following a critical/destabilizing injury such as anterior cruciate ligament (ACL) rupture (see Blaker et al., 2021 and references therein) (271).

## Harnessing Monocyte and Macrophage Biology for OA Risk Stratification, Diagnosis, and Therapy?

It is now well recognized that ptOA development features an early inflammatory response. This involves systemic processes resulting in monocyte recruitment, as well as a local disbalance within the immune “niches” of the affected joint, whose immune privilege is therefore compromised. This recognition has several implications that in the future could be exploited for prognostic, diagnostic and therapeutic benefit, examples of which we discuss below.

### Monocytes and Macrophages as Biomarkers for OA

To this day, OA diagnosis largely depends on clinical presentation/symptoms and conventional imaging methods like x-ray, computerized tomography (CT) scans or magnetic resonance imaging (MRI) (272). Diagnostic biomarkers are currently missing, as are reliable predictive markers. Access to synovial fluid and hence, the search for useful biomarkers, is limited by the invasive nature of the acquisition procedure. Nonetheless, advances have been made recently in the search for cellular and molecular biomarkers with diagnostic and/or predictive potential (221, 273), using synovial fluid where available, or peripheral blood, which can be more readily obtained.

On the cellular level, a growing body of data implicates monocytes and monocyte-derived macrophages in OA pathogenesis, as discussed in this review. Several recent clinical studies therefore investigated the prognostic value of peripheral immune cell ratios. While the exact ratios differ between studies, monocytes represent a common denominator. In particular, the neutrophil to monocyte ratio is independently and inversely associated with OA severity as classified using the Kellgren-Lawrence scale (274). Similarly, the monocyte to lymphocyte ratio reliably predicts OA progression (275). MicroRNA analysis of peripheral blood mononuclear cells (PBMCs) from OA patients showed elevated expression of miRNA-146a and miRNA-155 (276), which influence inflammatory cell signaling *via* the NF-κB pathway (277, 278). Moreover, transcriptomic analysis of PBMCs from OA patients identified more than 1000 differently expressed genes, pathway analysis of which implicated inhibition of chondrocyte differentiation, increased osteoclastogenesis and MAPK activation (279). These data collectively indicate that peripheral blood monocytes of OA patients differ from healthy controls both quantitatively and qualitatively. It is tempting to speculate that specific OA-primed inflammatory monocytes exist in the peripheral blood during disease progression, and even potentially prior to onset. This notion is corroborated by data from Loukov and colleagues, who demonstrated that following *in vitro* exposure to DAMPs, peripheral blood monocytes from women with knee OA produced higher levels of the pro-inflammatory cytokines IL-1β and TNF-α than monocytes from healthy controls (280). The same group also demonstrated significantly higher levels of CD14 expression on monocytes of OA patients, further implicating non-classical activated monocytes.

Within the synovial fluid of knee OA patients, monocytes and macrophages constitute the second most abundant cell population after T cells, and a large proportion of these are CD16^-^, thus further implicating non-classical monocytes (281). Liu et al. investigated the relative abundance of phenotypically distinct macrophages in synovial fluid of knee OA patients and found an increased ratio of “classically” compared to “alternatively” activated macrophages (282). This ratio further correlated with disease severity, suggesting that despite the limitations of this simplistic dichotomy, such analyses can yield clinically relevant data.

A wealth of experimental and clinical studies has analysed inflammatory parameters and markers in synovial fluid for their potential to serve as biomarkers. These studies have shown that levels of CCL2, IL-6 and IL-8 accurately distinguish OA from normal joints (283–285) and inflammatory markers can even predict the outcome of ACL reconstruction. Similarly, the presence and severity of synovitis following meniscal injury are associated with the risk of progressive cartilage damage, even if inflammation subsequently resolves (286). Elevated levels of several additional synovial fluid biomarkers associate not only with radiographic OA severity (sVCAM-1, sICAM-1, TIMP-1 and VEGF) and OA symptoms (VEGF, MMP-3, TIMP-1, sVCAM-1, sICAM-1 and MCP-1) but are also highly correlated with levels of neutrophil elastase (287). This highlights a potential role for neutrophil activation in the onset of OA. These initial findings were further corroborated by a recent study indicating that expression levels of TGF-ß1 and elastase were associated with radiographic severity scores and predictive of knee OA progression (288).

Based on such findings, Jayadev *et al.* used a novel machine learning approach to develop a “cytokine fingerprint” for end-stage OA. Using a panel of eight biomarkers (PIIANP, TIMP-1, ADAMTS-4, CCL2, IP-10 and TGF-β3), this model distinguishes between OA, knee injury and inflammatory knee arthritis (i.e. RA or psoriatic arthritis) with almost 100% efficacy (289). Interestingly, knee/hip arthroplasty further increases the levels of angiogenic and pro-inflammatory cytokines, but leaves anti-inflammatory cytokines unaffected, suggesting underlying changes specifically in pro-inflammatory pathways, which might be further exacerbated with surgical treatment (290).

In summary, biomarker research has both leveraged and fueled the notion that monocytes contribute to OA pathogenesis, and that OA-primed non-classical monocytes might exist. Although this progress is encouraging, the majority of recently identified biomarkers are associated with disease progression, rather than onset. There remains thus a pressing, unmet clinical need for biomarkers instrumental in diagnosis and stratification of individuals at risk of developing ptOA following knee injury.

### Disease Modifying Drugs for OA: Where Are We on the Clinical and Pre-Clinical Level?

To target the early inflammatory phase that follows joint injury and initiates OA pathogenesis, adjuvant DMDs are urgently needed. A variety of agents with the potential to serve as DMDs are currently being tested, but unfortunately, with limited success (291). Some studies on local inhibition of IL-1 in animal models showed promising results. In particular after closed articular fracture, intra-articular injection of an antagonist for IL-1 receptor (IL-1Ra) reduces post-traumatic OA, cartilage degeneration and synovitis (292–294). However, the clinical efficacy of this approach remains controversial. While an early clinical study found that intra-articular administration of IL-1Ra performed within 1 month of severe knee injury led to reduced knee pain and improved function over a 2-week follow-up period (295), other clinical studies using either IL-1Ra (Anakira) or a dual variable domain immunoglobulin that simultaneously inhibits IL-1α and IL-1β (Lutikizumab) have yielded no benefit in OA patients compared to placebo controls (291). Interestingly, retrospective secondary analysis of a large-scale clinical trial of a monoclonal antibody targeting IL-1β for cardiovascular disease found reduced incidence of hip and knee replacement in patients with high C-reactive protein, suggesting a potential effect on OA progression in an inflammatory setting (296).

It is noteworthy, that the OA-specific studies of DMD candidates described above were tested mostly in patients with advanced OA with the intention of reducing established clinical and radiological disease progression. In line with the current difficulties in risk stratification and early diagnosis outlined above, to date, no drugs have been advanced to the clinical stage that target the inflammatory response at the onset or early stages of disease. Experimentally, however, attempts to advance in this direction have been made recently. One study explored the role of incretin hormone receptors *in vitro*. It found that an analogue for the human Glucagon-Like Peptide-1 (GLP-1), liraglutide, reduced production of reactive oxygen species, IL-6 and CCL2, reduced collagen and aggrecan degradation and inhibited inflammation *via* deactivation of NF-kB signaling (297). Similarly, administration of dexamethasone, rapamycin or BMP-7 results in a more anti-inflammatory macrophage phenotype *in vitro*. Whilst these findings await *in vivo* confirmation, these drugs might hold potential of modulating synovial inflammation in patients (298).

One of the few *in vivo* studies investigating the impact of anti-inflammatory therapy immediately after joint injury utilized a porcine model. Here, the authors induced OA *via* transection of the anterior cruciate ligament (ACL) and immediately provided corticosteroids by intraarticular injection, which resulted in mitigated collagen degradation, reduced monocyte recruitment and a less inflammatory macrophage profile/phenotype (299). Our group has investigated the respective effects of intraarticular administration of hyaluronan or BM-derived mesenchymal stem cells in a murine OA model. We found that hyaluronan therapy increased anti-fibrotic macrophages and decreased pain sensitization, while treatment with MSCs did not impact pain, but led to long-term chondroprotection (300). Along similar lines, another recent study demonstrated that administration of Alpha defensin-1 renders macrophages less inflammatory and attenuates OA in a surgical model (301). Collectively, these data suggest that specific anti-inflammatory treatment immediately after knee injury might represent a promising future therapeutic approach, and thus, justify additional experimental and ultimately clinical studies.

## Concluding remarks, Open Questions, and Future Directions

### Distinct Inflammatory Responses in Different Joint Injuries?

Post-traumatic OA accounts for nearly 12% of all cases of symptomatic OA (302) and a recent longitudinal cohort study showed that the risk of developing OA is almost sixfold increased by knee injury at a young age (303). Further stratification of these data revealed distinct risks for different injury types: ACL injury, meniscal tears and articular fractures of the tibia (risk difference (RD) of 19.5%, 10.5% and 6.6%, respectively) were associated with the highest risk (303). In trying to identify possible reasons for these differences, the simplest explanation of variable biomechanical aberration is insufficient, as an abundance of data has shown that restoration of biomechanics alone does not prevent ptOA development (4). An alternative hypothesis is that phenotypic differences exist in the pathogenesis of ptOA depending on the type of tissue affected by injury. Thus, metabolic and immunobiological differences may determine the individual risk of developing ptOA. Although comprehensive studies investigating the tissue- and phenotype-specific immune response after joint injury are missing, existing evidence supports an immunological role for the meniscus (304), which engages in a pro-inflammatory crosstalk with the synovium in OA (305). Furthermore, synovial fluid, cartilage tissue and isolated cartilage cells display distinct pro-inflammatory cytokine profiles depending on the type of pathology, further supporting the notion that a phenotype-specific cytokine topography exists in the joint (306). Future studies should therefore be designed to examine the inflammatory reaction associated with different types of joint injury.

### Could Restoring Joint Immune Homeostasis Hold the Key to OA?

Owing to decades of research, we have come to understand that OA cannot simply be attributed to “wear and tear” resulting from biomechanical changes. Rather, OA results from a complex biological response of different cells in multiple joint tissues, with “inflammation” playing a crucial role this process. Monocytes and macrophages are emerging as key players in the inflammatory process associated with OA. Circulating monocytes are particularly attractive as druggable targets, but selective targeting of tissue-resident macrophages might be equally feasible, for example using antibody-conjugated lipid nanoparticles (72). It is tempting to speculate that dysregulated dynamics between monocytes, macrophages and other cell types they cross-react with in the joint not only fuels pathology, but in fact represents an underlying cause of OA. To harness these intricate cellular interactions for diagnostic and therapeutic purposes, we need to further improve our understanding of monocyte and macrophage biology in both healthy and arthritic joints. Deciphering their developmental and functional dynamics harbours the potential of one day being able to restore synovial immune homeostasis and thus, finally provide a causative treatment for this debilitating disease.

### Can Findings From ptOA Be Translated to Other OA Phenotypes?

A wide range of small and large animal models for OA have been developed, and these have recently been reviewed (307). Far and away the most commonly used models are those induced by joint injury. They share a comparably rapid onset and highly standardized disease progression, and allow investigation of underlying molecular and cellular mechanisms, as well as evaluation of potential treatments at different stages of disease progression. However, the translation of findings made in these models to clinical settings has remained challenging (308). One explanation is the current discrepancy between preclinical research predominantly using ptOA models and clinical studies, the majority of which investigate late-stage “primary OA” which occurs in the absence of prior trauma or disease (309). There is emerging evidence that the pathophysiologic mechanisms of structural and symptomatic OA differs depending on the key initiating factors or disease phenotype (310, 311). How different the complex cellular inflammatory response is in different OA phenotypes remains to be resolved. In a first attempt to overcome this issue, findings from preclinical ptOA studies should be tested in preclinical models of primary OA, such as spontaneous age-associated and metabolic/obesity-induced disease. Whilst the associated immune response may be different in strength and spatio-temporal patterns, it is likely that at least some aspects of monocyte and macrophage biology relevant to ptOA apply to other OA phenotypes, such as their roles in ECM degradation and chondrocyte death, which are universal OA disease features. Likewise, some molecular signals identified in ptOA models as regulators of macrophage activation and polarization, such as specific cytokines and chemokines, and are also present in multiple OA phenotypes (312).

### Open Questions and Future Directions for Research

- Does the initial inflammatory response following joint injury pathologically imprint monocytes, macrophages and their stromal niche in the joint?
- Do disease-specific, “imprinted” populations of monocytes and macrophages emerge prior to disease onset? Do they mediate (or: propagate) OA pathogenesis?
- Are monocyte and macrophage dynamics permanently altered following joint injury?
- Can these populations be targeted therapeutically?
- Is there an optimal ratio between pro- and anti-inflammatory monocyte/macrophage subsets that mitigates the risk of OA after joint injury?
- Can a threshold be determined that governs the future direction of either resolution of inflammation and restoration of joint function or ongoing inflammation that contributes towards development of OA?
- Do similar considerations also apply to non-traumatic forms of OA?

Addressing these questions will provide the critical scientific understanding necessary to improve diagnosis, risk prognosis, and underpin development of specific targeted therapies to prevent OA onset and/or slow its progression following joint injury. Recognizing that the targets may differ depending on injury and change with time and being able to identify these therapeutic stages/windows, will be key to providing effective individualized patient management.

## Author Contributions

Conception and design of study: PH, CL, BM, and RG. Acquisition of data: PH, RG, CL, and MP. Drafting of article: PH, RG, MP, CL, and BM. Revising it critically for important intellectual content: PH, RG, MP, CL, and BM. All authors have approved the final version of the manuscript.

## Funding

The authors acknowledge salary support from the following agencies: Raymond E Purves Foundation PhD Scholarship (PH), University of Edinburgh and the Kennedy Trust for Rheumatology Research (RG, MP). The funding bodies had no input into the design, drafting, editing or content of the manuscript.

## Conflict of Interest

The authors declare that the research was conducted in the absence of any commercial or financial relationships that could be construed as a potential conflict of interest.

## Publisher’s Note

All claims expressed in this article are solely those of the authors and do not necessarily represent those of their affiliated organizations, or those of the publisher, the editors and the reviewers. Any product that may be evaluated in this article, or claim that may be made by its manufacturer, is not guaranteed or endorsed by the publisher.
